# Supplementary material for: Effect of crocin and treadmill exercise on oxidative stress and heart damage in diabetic rats
Source: PLoS One. 2023 Dec 19;18(12):e0281692. doi: 10.1371/journal.pone.0281692 (PMC10729987; doi:10.1371/journal.pone.0281692)
Supplement: Attachment — Submitted filename: Comment-R2.docx [file pone.0281692.s001.docx]

**Dear Editor-in-Chief;**

**Ref: Submission ID PONE-D-23-00007R1**

We thank the editor and reviewers for the time taken to conduct their reviews and the thoughtful feedback they provided. We have actioned/responded to the editor and reviewer feedback below and in the revised manuscript using the yellow background function. The authors have carefully considered the comments and tried our best to address every one of them.

In conclusion, we are pleased to see considerable support for our study and believe the paper is improved due to these changes. We believe the manuscript will interest your readership and audience and look forward to hearing from you in due course regarding this revision.

Yours sincerely;

Journal Requirements:

Please review your reference list to ensure that it is complete and correct. If you have cited papers that have been retracted, please include the rationale for doing so in the manuscript text, or remove these references and replace them with relevant current references. Any changes to the reference list should be mentioned in the rebuttal letter that accompanies your revised manuscript. If you need to cite a retracted article, indicate the article’s retracted status in the References list and also include a citation and full reference for the retraction notice.


Reviewers' comments:

Reviewer's Responses to Questions

**Comments to the Author**

1. If the authors have adequately addressed your comments raised in a previous round of review and you feel that this manuscript is now acceptable for publication, you may indicate that here to bypass the “Comments to the Author” section, enter your conflict of interest statement in the “Confidential to Editor” section, and submit your "Accept" recommendation.

Reviewer #1: (No Response)

Reviewer #2: (No Response)

2. Is the manuscript technically sound, and do the data support the conclusions?

The manuscript must describe a technically sound piece of scientific research with data that supports the conclusions. Experiments must have been conducted rigorously, with appropriate controls, replication, and sample sizes. The conclusions must be drawn appropriately based on the data presented.

Reviewer #1: Yes

Reviewer #2: Yes

3. Has the statistical analysis been performed appropriately and rigorously?

Reviewer #1: Yes

Reviewer #2: Yes

4. Have the authors made all data underlying the findings in their manuscript fully available?

Reviewer #1: No

Reviewer #2: No

5. Is the manuscript presented in an intelligible fashion and written in standard English?

Reviewer #1: No

Reviewer #2: Yes

6. Review Comments to the Author

AUTHOR RESPONSE:

**Reviewer #1:** Dear Dr. Hashemkandi,

I appreciate the paper and results you have presented. Although they are not entirely original, most of these insights have been described in previous publications by other research groups. However, the data provided here support and complement the previous findings. I particularly commend the simplicity and focus of the project, which was conducted entirely in vivo. The study contributes to our understanding of cardiovascular defects associated with diabetes and their contribution to the lethality in DM. It also explores new possibilities for patients to mitigate the impact of reactive oxygen species (ROS) on cardiac tissue, thereby increasing the potential to reduce risks. The authors should include references of important previous works in these areas. For instance, a study from the same center demonstrated beneficial effects of exercise on cardiovascular damage in diabetic rats (Naderi et al, 2015 Adv Pharm Bull). Additionally, previous studies have shown crocin, an antioxidant agent presents in saffron, reduces the impact of ROS in cardiovascular tissue in diabetics (Farshid et al, 2016, Avicenna J Phytomed). The findings of these papers should be discussed on the manuscript. Furthermore, it is essential to improve the English usage, grammar and vocabulary especially in the abstract. I agree that revised version is improved respect to the first one. Another general concern I have is regarding how the rat groups are described along the text. To enhance readability, I suggest using “diabetes group”, “diabetes treated group (exercise/crocin)” and “control/healthy group”, instead of “diabetes control group”. I also concur previous reviewer’s suggestion to provide access to the raw data. I highly recommend that the authors make it publicly available. Here some additional points that I believe should be addressed:

AUTHOR RESPONSE

Thank you for your comments. We have gone through your comments carefully and tried our best to address them one by one. We hope the manuscript has been improved accordingly.

1. I would like to know the exact number of rats in each of the 8 groups detailed in “Animal preparation and study design” section of Material and Methods.

Author's response:

Thank you for your comments. This study used 64 rats in 8 groups (n=8).

2. In table 3, 4, 5 and 6 please indicate when the samples for the measurements were collected.

Author's response:

Thank you for your comments. All measurements were done after the exercise and use of crocin.

3. For figures 1, 2 and 3, as well as tables 2 and 3, clarify the representation of
numbers/bars (average, median, …) and the type of error bars used (SEM, SE,..).

Author's response:

Thanks for your feedback. We clarified central tendency and dispersion measures in Tables 2 and 3. It also described the error bar type SD standard Deviation in Figures 1, 2, and 3. Revisions were highlighted in the figures and tables.

4. Include reference to previous studies that have been assessed the effects of crocin and exercise on generation of cardiovascular damage in diabetes models.

Author's response:

Thanks for your feedback. Your suggested studies were added and highlighted.

5. Consider improving the visual presentation of tables by using graphs, particularly for tables 5 and 6.

Author's response:

Thanks for your feedback. We clarified central tendency and dispersion measures in Tables 5 and 6. We observed that to show all data better, we must use table format.

6. In the histological studies, I highly recommend implementing a method to quantify the extent of histological damage. This way you can strengthen the analysis and provide more robust assessment of the observed histopathological changes. This also will enhance the objectivity and reliability of the findings and will complement the microscope images.

Author's response:

Thank you for your valuable feedback and suggestions regarding the histological studies in our manuscript. We appreciate your insightful recommendation to implement a method for quantifying the extent of histological damage, which could strengthen our analysis and provide a more robust assessment of the observed histopathological changes. We acknowledge the importance of quantification in histological studies to enhance objectivity and reliability while complementing the microscope images. However, we regret to inform you that, due to limited resources and current institutional constraints, we cannot access specialized software or tools that can effectively analyze H&E staining results for quantification purposes. Despite this limitation, we have taken several steps to address the issue and provide the most thorough analysis possible within our constraints. We have ensured that the histopathological assessments were conducted by experienced pathologists who followed established guidelines and employed a consistent grading system for the qualitative evaluation. While this may not provide quantitative data, it offers valuable qualitative insights into the observed changes. We have also included detailed descriptions and figures to illustrate the histological findings, allowing readers to assess the extent of damage visually.

Moreover, in the discussion section of the manuscript, we have emphasized the qualitative aspects of the histological results and their clinical implications. We understand that quantification would be a valuable addition to our study and appreciate your understanding of our limitations. We hope the comprehensive qualitative analysis we have provided and the other rigorous aspects of our research can still contribute meaningfully to the field.

Once again, we thank you for your constructive feedback, and we remain open to any further suggestions or comments you may have to improve the quality and impact of our manuscript.

7. It would be beneficial to include comments in the text regarding the recovery of kidney function after exercise and crocin treatment, supported by the levels of creatinine and urea. Additionally, I have some suggestions to enhance the comprehensiveness of the paper and introduce further novelty by incorporating complementary readouts to the experiments:

Author's response:

Thanks for your feedback. Your suggested studies were added and highlighted. About the suggested evaluation tests. First, we would like to thank you for your suggestion. But, since our study was completed at least 1 year ago, we cannot do your mentioned test.

1. In addition to measuring of MDA, SOD and GPx to asses oxidative stress, that is an indirect way, consider including another assay, there are a bunch of kits that help to confirm an increase of the oxidative stress, I will recommend to use one to check the oxidation levels of the heart proteins in the 8 groups of rats.

Author's response:

First, we would like to thank you for your suggestion. But, since our study was completed at least 1 year ago, we cannot do your mentioned test.

2. To complement the histological approach and confirm apoptosis in a direct way, consider conducting a functional assay, a very easy one will be to measuring annexin V levels by flow cytometry in rat heart cells. Additionally, to investigate apoptosis induction, which is determined by the balance of pro- and anti- apoptotic regulators, consider measuring the levels of other apoptotic regulators by qPCR (Bcl x, MCL1, BAD, BIM,…) this way results would be more robust. Alternatively, BH3 profiling offers a novel and interesting approach to test apoptosis. In conclusion, I find the work very promising, and with the necessary improvements, I would recommend that the editor accept it for publication in PLOS ONE.

Author's response:

First, we would like to thank you for your suggestion. But, since our study was completed at least 1 year ago, we cannot do your mentioned test.

Reviewer #2: Overall, the introduction provides a comprehensive overview of diabetes, its mechanisms, and potential treatments. However, there are some points that require further attention to ensure clarity, precision, and context. Here's a review of the introduction with suggestions for improvement:

AUTHOR RESPONSE

Thank you for your comments. We have gone through your comments carefully and tried our best to address them one by one. We hope the manuscript has been improved accordingly.

1. Clarity and Precision:
• In the sentence "Diabetes increases the apoptosis rate...", the direct causation between diabetes and apoptosis should be substantiated or rephrased to avoid overstatement.

AUTHOR RESPONSE

Thanks for your feedback. Your suggested sentence was rewritten and highlighted.

Diabetes increases the apoptosis rate (Pancreatic β-cell apoptosis is also a pathological feature common to Type 1 diabetes mellitus (T1DM) and T2DM. In T2DM, insulin resistance with visceral obesity leads to a glucose toxicity effect, which accelerates β-cell death by apoptosis) in heart cells and disrupts cardiac function

• In the last sentence, you mentioned "treatment with kerosene." It's a bit surprising because kerosene is generally known as a fuel. If there is a specific compound within kerosene that has therapeutic effects, it should be specified.

AUTHOR RESPONSE

Thanks for your feedback. Your suggested sentence was rewritten and highlighted.

Accordingly, this study aimed to determine the effect of forced treadmill exercise and concomitant treatment with crocin on heart tissue damage and oxidative stress in diabetic rats.

2. Citations:
• Ensure that the cited references correspond to the information given, and that there is no repetition in citations. For example, '(9)' is cited twice with different information.

AUTHOR RESPONSE

Thanks for your attention. Checked and corrected.

3. Structural Flow:
• Consider reordering the details on the mechanics of DM, antioxidants, and ROS for better flow.

AUTHOR RESPONSE

Thanks for your attention. Checked and corrected.

• The section about Crocin and crustin feels a bit out of place. It may be helpful to introduce why these compounds are relevant to the study, perhaps with a transition sentence.

AUTHOR RESPONSE

Thanks for your attention. It was checked, rewritten, and highlighted in the manuscript.

Crocin and crustin are the predominant carotenoids that play a pivotal role in determining the coloration of saffron. Crocin undergoes metabolic processes within the human body, forming Crocin, which exhibits numerous medicinal qualities.

4. Grammar and Wording:
• "...as high blood sugar interferes with the metabolism of fats, carbohydrates, and proteins." – This can be rephrased for clarity.

AUTHOR RESPONSE

Thanks for your attention. It was checked, rewritten, and highlighted in the manuscript.

Diabetes mellitus is a syndrome characterized by lipid, carbohydrate, and protein metabolism disruption due to elevated blood sugar levels. Consequently, it has the potential to heighten the susceptibility to vascular disease.

• In "Crocin and crustin are the most critical carotenoids responsible for the color of saffron.", it's unclear what "most critical" means. Does it mean they are the most abundant or most essential for the color?

AUTHOR RESPONSE

Thanks for your attention. It was checked, rewritten, and highlighted in the manuscript.

• The phrase "not yet exposed to DNA" is unclear. Do you mean an enzyme that hasn't interacted with DNA yet?

AUTHOR RESPONSE

Thanks for your attention. It was checked, rewritten, and highlighted in the manuscript.

About the suppressive impact of crocin on tumorigenesis, a plausible hypothesis may be formulated suggesting that crocin functions as a stimulator for a DNA-repair enzyme that has not yet encountered DNA.

5. Scientific Accuracy:

• "Apoptosis, or programmed cell death, is a natural and active process during evolution after cells are exposed to cytotoxic agents." – Apoptosis is a cellular process that can occur for various reasons, not just exposure to cytotoxic agents. The mention of evolution here is also a bit confusing.

AUTHOR RESPONSE

Thanks for your attention. It was checked, rewritten, and highlighted in the manuscript.

Apoptosis, also known as programmed cell death, is an inherent and dynamic biological process that occurs in response to exposure of cells to cytotoxic substances, as observed throughout evolution. Apoptosis is characterized by several prominent aspects, namely cellular shrinkage, membrane impairment, chromatin condensation, and fragmentation of DNA. Multiple proteins have a role in the regulation of apoptosis. The Bcl-2 protein family encompasses a group of proteins that exert regulatory control over apoptosis, playing pivotal roles in inhibiting and promoting this cellular process. While the Bcl-2 protein functions as a suppressor, the Bax protein serves as a promoter of apoptosis.

• "...oxidative stress induced by acute exercise can affect the erythrocytes of non-exercised rats" – If there's a distinction between "non-exercised rats" and "exercised rats", it should be clearer.

AUTHOR RESPONSE

Thanks for your attention. It was checked, rewritten, and highlighted in the manuscript.

It has been shown that oxidative stress induced by acute exercise can affect the erythrocytes of non-exercised rats" and "exercised rats, while it does not significantly affect the erythrocytes of exercised rats.
6. Relevance:

• The relationship between crocin, crustin, diabetes, and oxidative stress should be made more explicit since this seems to be the premise of the study.

AUTHOR RESPONSE

Thanks for your attention. It was entirely considered in the manuscript.

• The last sentence provides an objective for the study, but the connection between forced treadmill exercise, kerosene, and diabetes should be clearer.

AUTHOR RESPONSE

Thanks for your attention. It was checked, rewritten, and highlighted in the manuscript.

In summary, the introduction provides valuable information but can benefit from improved organization, clarity, and precision in presentation. Ensure the relevance of all the information to the main topic, and make connections between sections explicit for better comprehension.
Materials and Methods provides a comprehensive insight into the methodology employed for the study. However, I would like to make a few suggestions and corrections to enhance clarity and precision.

AUTHOR RESPONSE

Thank you for your comments. We have gone through your comments carefully and tried our best to address them one by one. We hope the manuscript has been improved accordingly.

1. Clarity on Animal Preparation:
• Clearly specify how long after inducing diabetes the treatments (like exercise and crocin administration) started.

AUTHOR RESPONSE

Thank you for your comments. One week after the STZ injection, rats exhibiting hyperglycemia were considered type 1 diabetic and subjected to subsequent experiments.

• In the section detailing the groups, it's mentioned that a group was treated with kerosene, but there's no mention of kerosene in the subsequent details. Ensure there's consistency in the narrative.

AUTHOR RESPONSE

Thank you for your comments. Our goal was crocin, so the corrected word was replaced.

2. Consistency with References:
• Ensure that all the references are consistently numbered and are in sequential order. For instance, there's a jump from (10) to (11) without any (11) mentioned.

AUTHOR RESPONSE

Thank you for your comments. All of the references were double-checked.

3. Technical Corrections:
• Ensure you maintain consistency in your units and representations. For instance, the speed “10/55-83 m/min” is not clear. This probably needs correction.

AUTHOR RESPONSE

Thank you for your comments. The required part was corrected.

• In the sentence, "blood glucose levels were measured by an EASY GLOCO device once a day at a 50 mg/kg dose by gavage," it's not clear what the "50 mg/kg dose by gavage" refers to. Was this the frequency of measuring glucose levels or related to the administration of streptozotocin or crocin?

AUTHOR RESPONSE

Thanks for your attention. It was checked, rewritten, and highlighted in the manuscript.

After 72 hours, blood glucose levels were measured by an EASY GLOCO once daily. Then, crocin at a 50 mg/kg dose by gavage to the rats.

4. Biochemical Analysis:
• When you mention “based on our previous study”, it’s crucial to provide a reference for that particular study so readers can trace back if needed.

AUTHOR RESPONSE

Thanks for your attention. It was checked, and required reference was added.

5. Quantitative real-time polymerase chain reaction (RT-qPCR):

• It would be beneficial if you could provide a bit more detail on the thermal cycling conditions (denaturation, annealing, and extension temperatures and times).

AUTHOR RESPONSE

Thanks for your attention. We provided details of denaturation, annealing and temperature and times for RT-aPCR in text and highlighted by yellow background under (Methods, Quantitative real-time polymerase chain reaction (RT-qPCR, line …).

6. Statistical Analysis:

• For a more robust statistical inference, mention if any post-hoc test (e.g., Tukey, Bonferroni) was used after the ANOVA.

AUTHOR RESPONSE

Thanks for your attention. SPSS software (version 21.0, Chicago, IL, USA) was used for data analysis. One-way analysis of variance (ANOVA) followed by a multiple two-tailed T-test was used to study significant differences among the collected data. P <0.05 was considered statistically significant.
7. General Writing:

• Some sentences are overly complex and might benefit from being split or restructured for clarity.

AUTHOR RESPONSE

Thanks for your attention. General Writing of the manuscript was double-checked.

• Ensure that abbreviations are consistently used after being introduced the first time.

AUTHOR RESPONSE

Thanks for your attention. All of them were double-checked.

8. Histopathological evaluation:

• When you mention the rat heart tissue sections were "thoroughly scrutinized by a light microscope (Nikon) by two independent judges in a blindfold condition," consider using "blinded condition" or "double-blinded method" instead of "blindfold condition" for clarity.
Remember, the Materials and Methods section should offer enough detail so that another researcher could replicate the study. Ensure every step, condition, and protocol is described comprehensively.

AUTHOR RESPONSE

Thanks for your attention. It was corrected and highlighted in the manuscript.

Material methods were rechecked.

Result

Histology figure in your results section lacks resolution, this can be a significant concern. Clear, high-resolution images are essential for readers and peer reviewers to make accurate assessments of your findings. Here's what you can consider doing:

1. Re-scanning or Re-imaging:

• If you still have access to the original histological slides, consider re-scanning or re-imaging them using a high-quality scanner or microscope equipped with a camera. Ensure that you're using the highest possible settings to capture the best resolution.

Authors response:

Thanks for your comment. We quantified the level and/or value of the histological damage based on the study groups as shown in Figure 4 (microscope images).

2. File Format:

• Save the image in a lossless file format like TIFF or PNG. These formats preserve image quality better than JPEG, which can lose some data every time the image is saved.

Authors response:

Thanks for your comment. All of the figures were saved in TIFF format.
3. Compression:
• Avoid compressing the image. Compression can reduce the image's quality and resolution.

Authors response:

Thanks for your considerable comment.

4. Figure Annotation:

• If annotations (like labels, arrows, etc.) were added to the image, ensure that the annotations themselves are clear and sharp. Use software that allows vector-based annotations, which won't degrade upon resizing.

Authors response:

Thanks for your considerable comment.

5. Image Enhancement:

• If the image appears a bit unclear due to issues like low contrast, consider using image editing software to adjust the contrast, brightness, or sharpness. However, be very cautious; any modifications should be ethical, and they shouldn't change the underlying data or mislead the viewer. Always mention any enhancements made in the figure caption or materials and methods section.

Authors response:

Thanks for your considerable comment.

6. Consider Alternative Representation:

• If the tissue stain or preparation was not clear, and you're unable to capture a high-resolution image, you might want to consider an alternative method to represent the histological findings. For example, a schematic or illustration might be used to complement the original figure. However, the original, unaltered image should always be provided.

Authors response:

Thanks for your considerable comment.

7. Figure Caption:

• Ensure the figure caption provides sufficient details about the image. Mention the magnification, stain used, and any other pertinent details.

Authors response:

Thanks for your considerable comment. It was considered.

8. File Size Limitations:

• Some journals have limits on the file size of images. If your high-resolution image exceeds the journal's size limit, reach out to the editorial team. They might have provisions or suggestions for including large files.

Authors response:

Thanks for your considerable comment. It was considered.

Lastly, always double-check how the image looks in the manuscript, both on screen and printed. This will give you an idea of the clarity and quality that your readers will experience.

AUTHOR RESPONSE

Thank you for your comments. We have gone through your comments carefully and tried our best to address them one by one. We hope the manuscript has been improved accordingly.
